# Developing practical clinical tools for predicting neonatal mortality at a neonatal intensive care unit in Tanzania

**DOI:** 10.1186/s12887-021-03012-4

**Published:** 2021-12-01

**Authors:** Dory Kovacs, Delfina R. Msanga, Stephen E. Mshana, Muhammad Bilal, Katarina Oravcova, Louise Matthews

**Affiliations:** 1 Boyd Orr Centre for Population and Ecosystem Health and Institute of Biodiversity, Animal health and Comparative Medicine, University of Glasgow, Glasgow, G12 8QQ UK; 2 Department of Paediatrics and Child Health, Catholic University of Health and Allied Sciences, Mwanza, Tanzania; 3 Department of Microbiology and Immunology, Catholic University of Health and Allied Sciences, Mwanza, Tanzania; 4 Quality Operations Laboratory, University of Veterinary and Animal Sciences, Lahore, Pakistan

**Keywords:** Early warning systems, LMIC, Machine learning, Neonatal mortality, Vital signs

## Abstract

**Background:**

Neonatal mortality remains high in Tanzania at approximately 20 deaths per 1000 live births. Low birthweight, prematurity, and asphyxia are associated with neonatal mortality; however, no studies have assessed the value of combining underlying conditions and vital signs to provide clinicians with early warning of infants at risk of mortality. The aim of this study was to identify risk factors (including vital signs) associated with neonatal mortality in the neonatal intensive care unit (NICU) in Bugando Medical Centre (BMC), Mwanza, Tanzania; to identify the most accurate generalised linear model (GLM) or decision tree for predicting mortality; and to provide a tool that provides clinically relevant cut-offs for predicting mortality that is easily used by clinicians in a low-resource setting.

**Methods:**

In total, 165 neonates were enrolled between November 2019 and March 2020, of whom 80 (48.5%) died. We competed the performance of GLMs and decision trees by resampling the data to create training and test datasets and comparing their accuracy at correctly predicting mortality.

**Results:**

GLMs always outperformed decision trees. The best fitting GLM showed that (for standardised risk factors) temperature (OR 0.61, 95% CI 0.40–0.90), birthweight (OR 0.33, 95% CI 0.20–0.52), and oxygen saturation (OR 0.66, 95% CI 0.45–0.94) were negatively associated with mortality, while heart rate (OR 1.59, 95% CI 1.10–2.35) and asphyxia (OR 3.23, 95% 1.25–8.91) were risk factors. To identify the tool that balances accuracy and with ease of use in a low-resource clinical setting, we compared the best fitting GLM with simpler versions, and identified the three-variable GLM with temperature, heart rate, and birth weight as the best candidate. For this tool, cut-offs were identified using receiver operator characteristic (ROC) curves with the optimal cut-off for mortality prediction corresponding to 76.3% sensitivity and 68.2% specificity. The final tool is graphical, showing cut-offs that depend on birthweight, heart rate, and temperature.

**Conclusions:**

Underlying conditions and vital signs can be combined into simple graphical tools that improve upon the current guidelines and are straightforward to use by clinicians in a low-resource setting.

## Background

The world has made substantial progress in tackling infant mortality over the past 50 years [1], and neonatal (up to 28 days of age) mortality rates (NMR) are steadily falling in high-income countries (HICs). Of 2.4 million neonatal deaths in 2019 worldwide, the World Health Organization (WHO) [2] estimated that most occurred in low- and middle-income countries (LMICs). In Tanzania, NMR is falling at a much lower rate than mortality among children under 5 years [3] and mortality is especially high in preterm neonates [4]. Tanzania has made substantial efforts in improving neonatal care and reduce mortality by implementation of various guidelines and policies recommended by WHO. This includes essential new-born care, kangaroo mother care, integrated management of childhood illness (IMCI), growth monitoring, and care for childhood development. The national coverage of these interventions remains low (40%) compared to that recommenced by WHO (80%). To address the gap the government has consolidated national guidelines, which are available in all levels of health facilities. While improved thermal regulation of neonates using incubators, better formulas for nutrition, surfactants to treat respiratory distress syndrome, and antimicrobials to fight infections have significantly reduced new-born deaths in HICs, such as the UK [1], these are often unavailable or unaffordable to families in low-resource settings. Limited human resource and health systems capacity are factors that prevent the scale up of health interventions in LMICs [5]. Indeed, the main causes of neonatal deaths include prematurity, intrapartum-related complications, such as birth asphyxia, and preventable and treatable infections, such as early- and late-onset neonatal sepsis [2, 6]. Reducing the mortality of neonates remains an urgent challenge [7].

The United Nations Children’s Fund (UNICEF, [8]) estimated 19 deaths per 1000 live births in Tanzania in 2015, primarily caused by birth asphyxia (29.3%), prematurity (24.7%) and sepsis (19.7%). Two large studies in hospitalised neonates have also found the major causes of neonatal mortality to be birth asphyxia, preterm deliveries, infections (sepsis), respiratory distress syndrome and congenital malformations [9, 10]. However, no study has explored the relationship between mortality, causes of mortality and changes in vital signs, such as temperature or heart rate. These data are crucial when drafting policy recommendations regarding management of neonatal conditions, such as neonatal sepsis, and reducing mortality.

Early warning systems that indicate patients at high risk or with deteriorating conditions are increasingly recognised as valuable tools for clinicians [11]. Although such systems are used in medicine for adults and children, they are not well-developed for neonatal care, even in high-resource settings, with existing tools often complex to use, demanding in terms of data needed or specific to certain infant groups [12, 13]. A review of early warning- and track and trigger systems used for infants by Mortensen et al. [13] found that no such scoring systems existed for neonates in NICU or those born prematurely. Mitchell et al. [12] explored the feasibility of using an early warning score for neonates in Kenya and concluded that such scores could be useful to identify at-risk neonates. However, they also highlighted the issues with limited data recording in low-resource settings, which the score relies on.

Currently, the guidelines typically used in neonatal medicine are based on cut-offs for individual vital signs [14, 15]. For example, low temperature (< 36.5 °C) is often considered an indicator for clinical deterioration, but it is not normally used in combination with other measurements, such as oxygen saturation or heart rate, to predict neonatal outcomes. The Apgar score is a simple evaluation system to assess neonatal health including five easily evaluated vital signs: appearance, heart rate, reflexes, muscle tone, and respiration [16]. A tool that combines vital signs (throughout hospitalisation) and other risk factors associated with mortality could aid effective clinical decision making.

There is a demand not only for early warning systems for neonatal care, but also for tools that are easily usable in low-resource settings [12]. Computer-based clinical decision support tools are increasingly used in healthcare and may involve statistical and artificial intelligence or machine learning approaches [17]. Machine learning can identify patterns in complex datasets, predicting clinical outcomes, and has recently been explored in other areas of medicine, including diagnostics and prescribing practices [18–21]. However, the most of these machine learning tools are a black box approaches that generate algorithms not readily interpretable by a clinician or usable without access to computational resource, limiting their usefulness in a low-resource setting. For this reason, clinician-friendly tools, such as generalised linear models or decision trees whose results can be shown graphically, should be considered. We hypothesise that tools that use a combination of models, rather than individual measurements, give a more accurate prediction of neonatal mortality. These tools would need an appropriate balance between accuracy and ease of use in a low-resource setting.

Here, we aimed to i) identify risk factors associated with neonatal mortality in the NICU in Bugando Medical Centre, Mwanza, Tanzania, ii) identify the most accurate GLM or decision tree for predicting mortality, and iii) provide a tool that provides clinically relevant cut-offs for predicting mortality that is easily used by clinicians in a low-resource setting.

## Methods

### Study design and setting

This was a prospective study conducted at Bugando Medical Centre (BMC) between November 2019 and March 2020. BMC is a referral, consultant, and university teaching hospital in Mwanza, with a catchment population of 14 million people in the Lake Zone. Neonates may be born here if the pregnancies are identified as high-risk or admitted following referral from a lower-tier hospital. The centre has 950 beds in total, with 15 cots in the NICU for severely ill patients. Parental consent was sought for participation, and information on neonatal health was obtained from the patients’ medical records.

### Study population inclusion and exclusion criteria

The study enrolled prospective neonates admitted at NICU and whose parents/guardians voluntarily consented to participate on their behalf. All neonates admitted to the neonatal unit were eligible. During the study period, 165 neonates were enrolled. Neonates admitted to NICU typically have low birthweight (< 2500 g), breath abnormally (outside 30–60 breaths/minute), have temperate below or above the healthy range of 36.5 °C to 37.5 °C or a fifth minute Apgar score below 7 and/or failure to establish spontaneous breathing after delivery [14]. Diagnosis of sepsis was based on risk factors, clinical signs and symptoms such as fluctuation in body temperature, difficult in breathing, hypoglycaemia,lethargy,convulsions, jaundice and others as documented on WHO Young Infant Study Group and its methodology paper [22]. Preterm neonates were defined as babies who were born alive under 37 completed gestation weeks [23]. Birth asphyxia in this setting was defined as failure to initiate or sustain spontaneous breathing at birth and/or a fifth minute Apgar score < 7.00 [14]. Clinically respiratory distress in preterm neonates was defined from clinical manifestation of abnormal pulmonary function and hypoxia presented with tachypnoea, nasal flaring, intercostal, subxiphoid and subcostal retraction, cyanosis, and decreased auscultated breath sound.

### Data collection

Socio-demographic and clinical characteristics of neonates were collected using a structured pre-tested questionnaire. Clinical information including diagnoses such as sepsis, prematurity, perinatal asphyxia, and congenital anomalies were obtained from patient medical records. Admitted neonates were kept on monitors which recorded oxygen saturation and pulse rate. Axillary temperature [24] was measured to every admitted new-born by using a digital thermometer (OMRON Health Care, Tokyo Japan). Random blood glucose (RBG) was measured using a glucometer (One Touch, United State), RBG above 2.6 mmol/dL was considered normal. Neonatal information obtained from patient files and questionnaires was anonymised and aggregated in MS Excel and analysed using R version 3.5.3 (2019-03-11). Only the first measurements were used for analyses as most neonates lacked subsequent data.

### Risk factor analysis using GLMs

We used generalised linear models (GLMs) to investigate risk factors associated with neonatal mortality (outcome variable; death or survival). Since the outcome variable was binary, we used the binomial family. The initial model included 12 variables as follows. Birthweight (kg), random blood glucose (mMol-1), oxygen saturation (%), temperature (degrees Celsius), heart rate (beats per minute), and respiratory rate (breaths per minute) were continuous variables. Sex (78 males, 87 females), mode of delivery (129 vaginal, 36 caesarean section), cot category (66 normal, 59 premature, 40 post-operative), asphyxia (41 present, 124 absent), sepsis (29 present, 136 absent), and respiratory distress syndrome (84 present, 81 absent) were categorical variables. Apgar scores were excluded due to a high proportion of missing data. The models were run using the observed and standardised (to have mean of 0 and variance of 1) variables to allow comparison of the relative importance of variables. A stepwise backwards model selection, using the *drop1* function, was used to obtain models with the lowest Akaike information criteria (AIC). In addition, we also examined models with fewer variables than the final GLM with the goal of developing a tool that is easily used without computer access.

### Decision tree models

We developed decision trees using the *rpart* package [25]. All variables included in the initial GLM were included in the decision trees. The trees were then pruned to avoid overfitting. The best-fitting pruned tree is the one with the lowest cross-validation error.

### Model validation and comparison

GLMs were compared visually using receiving operating characteristic (ROC, *ROCR* package [26]) curves and using the area under the curve (AUC). The performances of GLMs were compared to decision trees using accuracy (the number of correct predictions divided by the number of predictions). We refitted the decision tree and GLM to 5000 samples of training and testing datasets (80/20 split). Using this method, we competed pruned and unpruned decision trees with GLMs. For each testing dataset, the winning the model was the model with the highest accuracy. If multiple models had the same highest accuracy, one was randomly chosen to be the winner.

## Results

### Neonatal characteristics

Over the study period, 165 neonates were enrolled, of whom 78 (47.3%) were male. Mean gestational age was 33.4 weeks, and mean birthweight was 2.1 kg. Most births were vaginal deliveries (78.2%, *n* = 129). Neonatal incubators and ventilators were not available at this facility during the study period, but all preterm infants had kangaroo mother care [27] and early continuous positive airway pressure (CPAP) for those with respiratory distress syndrome. The most common reason for hospitalisation was low or very low birthweight (*n* = 88), followed by prematurity (*n* = 86), respiratory distress syndrome (*n* = 84), asphyxia (*n* = 41) and sepsis (*n* = 29) (Table 1); neonates often presented with a combination of these conditions. Four new-borns were exposed to HIV.

**Table 1:** Diagnoses in neonates by outcome (*n* = 165)

Documented diagnosis	Death (***n*** = 80)	Survival (***n*** = 85)	Total (***n*** = 165)
Low/very low birthweight	59 (73.8%)	29 (34.1%)	88 (53.3%)
Prematurity	58 (72.5%)	28 (32.9%)	86 (52.1%)
Respiratory distress syndrome	50 (62.5%)	34 (40.0%)	84 (50.9%)
Asphyxia	19 (23.75%)	22 (25.9%)	41 (24.8%)
Sepsis	11 (13.75%)	18 (21.2%)	29 (17.6%)

**Fig. 1:**
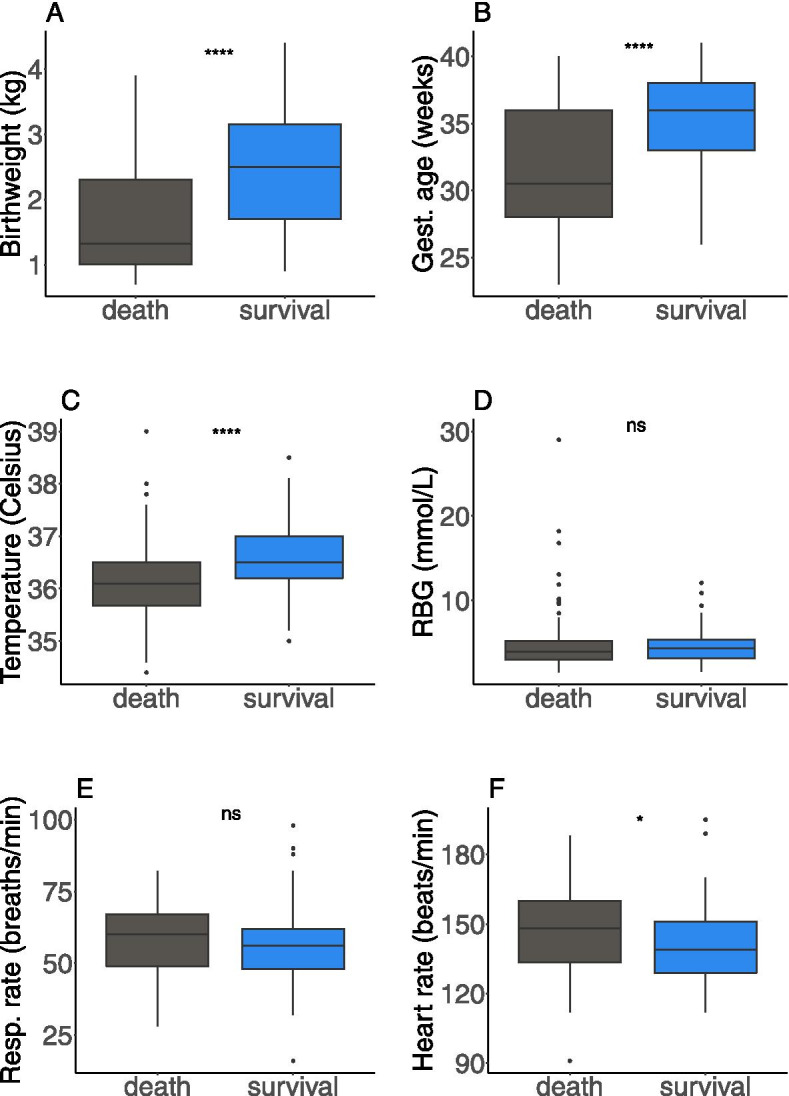
Neonatal characteristics by outcome (**A**) Birthweight (kg) (**B**) Gestational age (weeks) (**C**) Temperature (degrees Celsius) (**D**) Random blood glucose (mMol per litre) (**E**) Respiratory rate (breaths per minute) and (**F**) Heart rate (beats per minute). Ns = statistically non-significant difference

During the study period, the observed mortality was 48.5% (80/165) in the first 28 days of life, with the majority (78.8%, *n* = 63) of these occurring during the first week. Diagnoses of low or very low birth weight were more common in neonatal deaths (59/80) than survival (29/85) (Table 1). Similarly, prematurity was more frequently reported as the cause of hospitalisation in deaths (*n* = 58) than survivals (*n* = 28). Respiratory distress syndrome was also more common in neonates who did not survive (*n* = 50 vs *n* = 34). There were 29 cases of sepsis, 18 of which were diagnosed in neonates who survived. Out of the 41 asphyxia diagnoses, 22 occurred in surviving neonates. Neonates who died had a lower average birthweight (1.68 vs 2.44 kg), gestational age (31.7 vs 35.0 weeks), and temperature (36.2 vs 36.6 °C) than those who survived (Fig. 1) – these were all statistically significant (*p* < 0.0001). However, on average, they had a higher random blood glucose level (5.22 vs 4.70 mmol/L), respiratory rate (57.3 vs 56.3 breaths per minute) and heart rate (147 vs 141 beats per minute, *p* < 0.036).

### Risk factors associated with neonatal mortality obtained using a GLM

Following model selection, the GLM fitted to the full dataset with the lowest AIC (190.7) included birthweight, asphyxia, oxygen saturation, heart rate and temperature. The protective factors associated with mortality were temperature (OR 0.61, 95% CI 0.40–0.90), birthweight (OR 0.33, 95% CI 0.20–0.52) and oxygen saturation (OR 0.66, 95% CI 0.45–0.94). The risk factors associated with mortality were heart rate (OR 1.59, 95% CI 1.10–2.35) and birth asphyxia (OR 3.23, 95% CI 1.25–8.91) (Table 2). Odds ratios here refer to the standardised variables. For unstandardised variables, a one degree increase in temperature is associated with a reduction in mortality of approximately a half, a 1 kg increase in birthweight is associated with a reduction in mortality of approximately one third, a 1 % increase in oxygen saturation is associated with a reduction in mortality of 7%, and a one bpm increase in heart rate is associated with a 2% increase in mortality risk.

**Table 2:** Risk factors associated with neonatal mortality: odds ratios (ORs) associated with standardised and unstandardised variables

Variables	Covariate	Odds ratio	95% confidence interval
Standardised	Birthweight (kg)	0.33	0.199–0.518
	Asphyxia	3.23	1.251–8.912
	Oxygen saturation (%)	0.66	0.449–0.939
	Heart rate (bmp)	1.59	1.103–2.351
	Temperature (Celsius)	0.61	0.397–0.898
Unstandardised	Birthweight (kg)	0.30	0.178–0.495
	Asphyxia	3.23	1.251–8.912
	Oxygen saturation (%)	0.94	0.885–0.990
	Heart rate (bmp)	1.03	1.005–1.048
	Temperature (Celsius)	0.52	0.303–0.870

### Risk factors associated with neonatal mortality obtained using a decision tree

All variables from the initial GLM were included in the initial decision tree. The pruned tree (with minimised cross-validated error, including the complete dataset) and included birthweight only, predicting a cut-off of 1.325 kg. Using this cut-off, sensitivity was 50%, specificity 87% and accuracy 69%. Using this cut-off, 114 neonates were predicted to survive, and 64.9% (74/114) of them did. Among the remaining 51 neonates predicted to die, there were 40 (78.4%) deaths.

### Model validation and comparison

#### Comparing performances of decision trees and GLMs

We competed the pruned and unpruned decision trees against the final GLM (containing 5 variables) and also against simpler versions of the GLM that were the best fitting GLM with 4, 3, 2, and 1 variable(s). Using 5000 random splits of the data into training and test data, we showed that GLMs always outperform both pruned and unpruned decision trees when comparing based on accuracy.

#### Comparison of GLMs using ROC curves

**Fig. 2:**
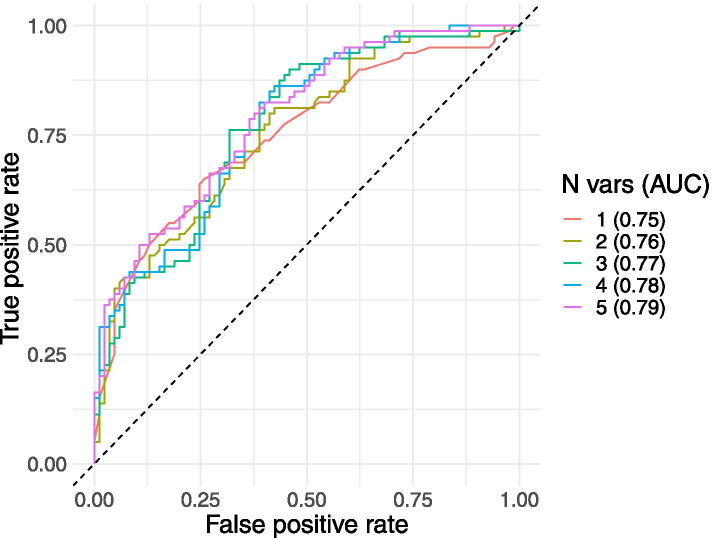
ROC curves (with areas under the curve, AUC) for GLMs fitted to the whole dataset with one to five variables, as follows: 1 – Birthweight; 2 – Birthweight and temperature; 3 – Birthweight, temperature, and heart rate; 4 – Birthweight, temperature, heart rate, and asphyxia; 5 – Birthweight, temperature, heart rate, asphyxia, and oxygen saturation

ROC curves were used to compare the five GLMs using the area under the curve (AUC) and to identify cut-offs for predicting mortality (Fig. 2). The one-variable GLM uses birthweight as the risk factor and has an AUC of 0.75, sensitivity of 65% and specificity of 74.1% (Table 3). Alternative single variable risk factors correspond to substantially lower AUCs: for temperature only, the AUC is 0.69; for heart rate only, the AUC is 0.61 and for O_2_ saturation only, the AUC is 0.6. Better performance was obtained when using risk factors in combination, with the AUC increasing from 0.75 for the birthweight only model, up to 0.79 for the GLM with all five risk factors: birthweight, temperature, heart rate, asphyxia, and oxygen saturation. GLMs with more than one risk factor also have higher sensitivity values compared to the GLM that includes birthweight only.

**Table 3:** Predicted cut-offs, with corresponding sensitivity and specificity values, accuracy, and area under the curve (AUC) for the final single and multiple variable models. Bpm = beats per minute

Risk factor(s) in GLM	Results					Existing cut-offs
	Predicted cut-off	Sensitivity	Specificity	Accuracy	AUC	
Birthweight	1750 g	65.0%	74.1%	0.69	0.75	Low birthweight < 2500 g, Very low birthweight < 1500 g
Temperature	36.5 °C	70.0%	63.5%	0.67	0.69	Local guidelines: Normal temperature 35.5 °C - 37.5 °C WHO guidelines: Normal temperature 36.5 °C - 37.5 °C
Heart rate	146 bpm	57.5%	67.1%	0.62	0.61	Bradycardia < 107 bpm Tachycardia > 170 bpm
O_2_ Saturation	91%	33.8%	81.2%	0.57	0.60	Cut-offs vary depending on gestational age and altitude.
Birthweight and temperature	–	81.3%	57.6%	0.70	0.76	–
Birthweight, temperature, and heart rate	–	76.3%	68.2%	0.72	0.77	–
Birthweight, temperature, heart rate, and asphyxia	–	86.3%	57.6%	0.72	0.78	–
Birthweight, temperature, heart rate, asphyxia, and O_2_ saturation	–	82.5%	60.0%	0.72	0.79	–

#### The final clinical tools

**Fig. 3:**
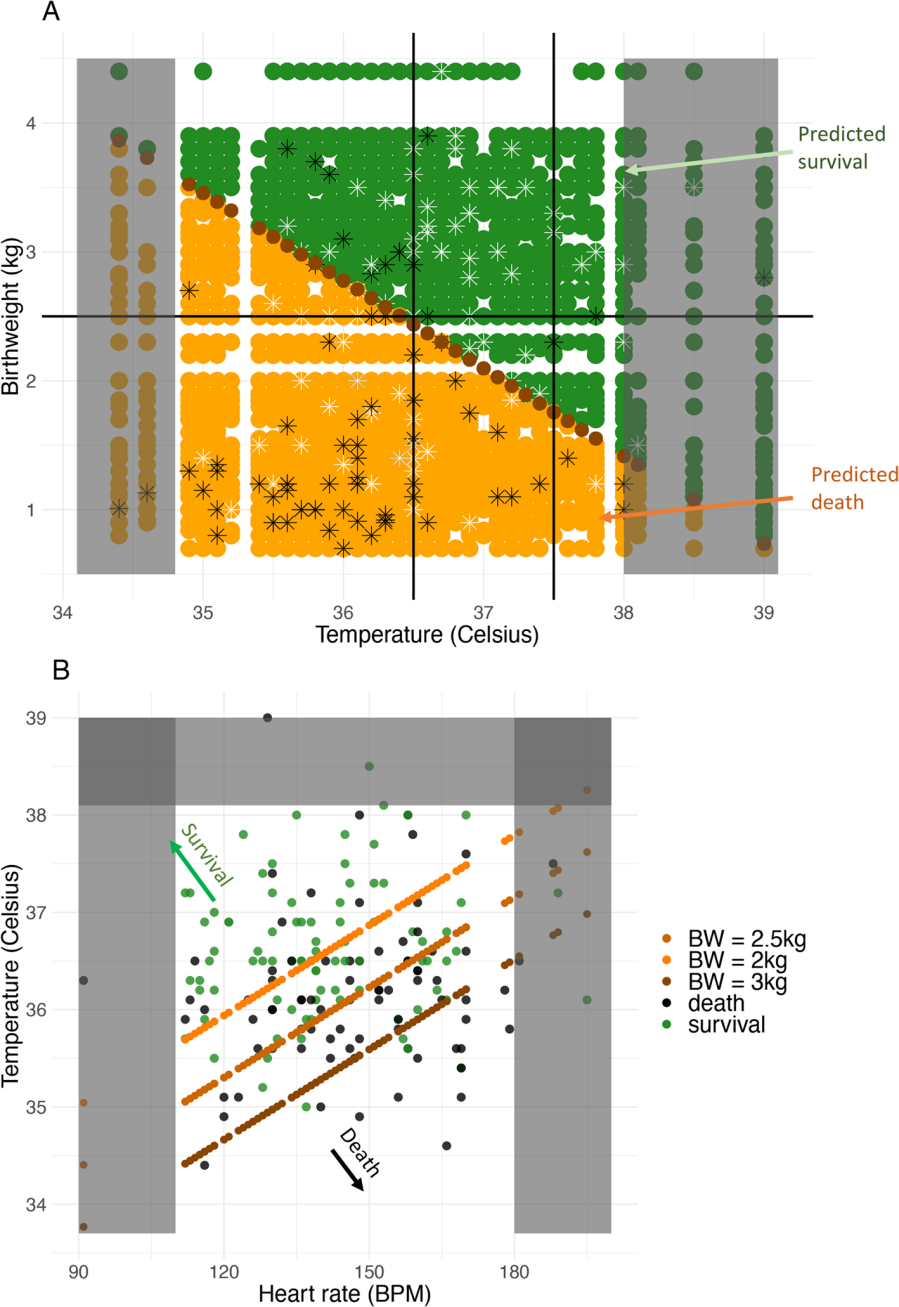
Final clinical tools.** A** Decision tool based on birthweight and temperature. Black lines represent WHO guidelines for normal birthweight (> 2500 g) and temperature (36.5–37.5 °C). Light orange area predicts death, green area predicts survival (10,000 simulated points). Dark orange line shows cut-off points, predicting survival for points above. Black stars represent observed deaths (n = 80), and white stars represent survivals (n = 85). **B** Decision tool based on temperature, heart rate and birthweight. Black dots represent observed deaths (*n* = 80), and white dots represent survivals (*n* = 85). Orange lines show different cut-offs based on birthweight. Infants above the cut-off line for their birth weight are predicted to survive (see arrows). For birthweights other than the three shown, selecting the cut-off associated with a lower birthweight is the conservative choice. For both tools, grey area shows temperature/heart rate extremes with few datapoints, not to be used

As GLMs outperformed decision trees, we used the GLMs models to develop a clinical tool for identifying neonates at risk of mortality. Whilst best performance based on accuracy or AUC is obtained for the five-variable GLM, the corresponding cut-off is difficult to present visually. We considered the two and three-variable GLMs as providing a good balance between performance and ease of presentation (Fig. 3). Figure 3A shows the values of temperature and birthweight for which mortality is predicted (light orange points) and those for which survival is predicted (green points). The cut-off line is shown in dark orange. For example, a neonate with 35.5 °C body temperature and 2.5 kg birthweight is predicted to be at risk of mortality, but a neonate with 37 °C temperature and 2.5 kg birthweight is predicted to survive. Three variables performed even better. Here, we present the cut-offs for three different values of the birthweights 2, 2.5, and 3 kg (Fig. 3B, orange points) with mortality predicted for points below the cut-off line and survival predicted for points above. For example, a neonate with 135 bpm heart rate, 36 °C temperature, and 3 kg birthweight is predicted to survive, but a neonate with 135 bpm heart rate, 36 °C temperature, and 2 kg birthweight is predicted to not survive. As few data points were recorded in neonates with very low or very high temperatures and heart rates, our tool should not be applied to such extremes.

## Discussion

Early warning systems in medicine are being increasingly adopted but remain insufficiently well-developed in neonatal medicine [13]. Moreover, there is a demand for early warning systems for neonatal mortality that are readily usable in low-resource settings [12]. Our research addresses the gap in availability of early warning systems for neonatal mortality that combine underlying conditions and vital signs and that are readily usable by clinicians in low-resource settings. Here, we used data from a cohort of neonates at Bugando Medical Centre, in Mwanza, Tanzania to first identify risk factors, including vital signs, for mortality and second, to use these findings to develop a potential clinical tool.

Using a standard statistical approach (GLMs), we identified the risk factors associated with neonatal mortality in neonates admitted to the NICU at BMC, including changes in vital signs. Low birthweight strongly associated with mortality – a finding consistent with previous studies [23]. Babies born preterm with low birth weight may die because of acute complications like hypothermia and hypoglycaemia [28–30], due to a lack of feasible, cost-effective care, such as breastfeeding, warmth support and basic care for breathing difficulties and infection. Low body temperature (defined as a core temperature below 36.5 °C) was also a risk factor. It is often associated with low birthweight and is known to increase mortality and morbidity [31]. Oxygen saturation and heart rate are vital signs that can change rapidly and are often monitored continuously. However, our analyses show that the first measurements (taken upon admission) were nevertheless significantly associated with mortality. Perinatal asphyxia, a condition known to account for much of neonatal mortality [31, 32], was also a significant risk factor.

Using this information, we considered the development of a tool for clinicians, to help identify neonates at risk of mortality. Due to their relative ease of interpretation, we compared GLMs and decision trees as potential tools that can be presented visually. Decision trees are flowcharts that have been used to aid classifications in medical problems [33–35] and could be used to formulate an early warning system, such as the one used in the UK to monitor deterioration of paediatric patients [11]. We then found that our final decision tree contained only birthweight, with a cut-off of 1.325 kg and an accuracy of 0.69. When comparing the accuracy of predictions across models, we found that the decision tree approach was inferior to a GLM, even when the GLM only included one variable (birthweight). More sophisticated machine learning tools such as random forests are likely to be more accurate but cannot be presented visually and therefore did not offer a viable option for our research.

Currently used guidelines in neonatal care typically focus on individual cut-offs but the inclusion of multiple variables improves accuracy. We used ROC curves and AUC to compare the performance of our binary classifier and obtain the cut-off corresponding to the sensitivity and specificity that optimises accuracy of classification. The best single risk factor in our GLM was birthweight, and using a ROC curve, we identified the cut-off to be at 1.750 kg with a corresponding accuracy of 0.69. Existing guidelines with a 2.5 kg cut-off correspond to a lower accuracy of 0.66. For temperature, the cut-off was 36.5 °C with an accuracy of 0.67, matching the WHO’s guidelines that define normal temperature range to be between 36.5 °C and 37.5 °C [15]. Accuracy increased when multiple variables were included in the model. For the best two-variable model (birthweight and temperature), accuracy was 0.70. Accuracy increased to 0.72 for the 3, 4 and 5 variable models. We selected the three-variable GLM (birthweight, temperature, and heart rate) as providing the best balance between accuracy and ease of use and showed an effective way of displaying the tool by plotting heart rate against temperature, with several cut-offs for different birthweights.

This tool could be effective in different settings but has been fine-tuned to fit our dataset obtained from a NICU with 48.5% mortality rate. It may require modification in other settings and should be supplemented with local data where possible. In addition, the trade-off between sensitivity and specificity must be kept in mind. A high sensitivity value, or the proportion of true positives, ensures that most neonates at risk of dying are detected. Given the seriousness of the outcome, it is important that at-risk neonates are not missed. However, a high number of false positives may put extra burden on healthcare staff and facilities in low-resource settings, and so desired specificity (which determines the number of false positives) should be considered.

Here, we presented a proof of concept for a simple tool that could be fine-tuned using additional data and ultimately lead to an evidence base that could be used to support policy recommendations around neonatal care. A larger sample size would improve accuracy of prediction and could also provide a fuller picture of risk at extremes of the measured variables where we had few datapoints available. Only the first set of recorded vital signs were used in this analysis, but incorporation of multiple measurements if available could also improve accuracy of predictions. Complete data on Apgar scores may also help to improve this tool.

The incompleteness of data underpinning our medical charts was a limitation of our study. The Apgar score is an important indicator of neonatal health, but it was not consistently recorded and therefore we excluded it from analysis. Our sample size was small, and we may have encountered a selection bias as enrolment relied on maternal consent.

Currently, there is a lack of early-warning systems for infant mortality in LMIC settings. A recent study in Kenya [12] demonstrated enthusiasm by stakeholders for an early warning scoring system for low birth weight or pre-term infants, with the ease of use of chart-based approach to capture multiple measurements being met with enthusiasm. We anticipate therefore that a decision tool based on our approach could be a useful addition to daily practice. Infants can be rapidly assessed against the chart whenever vital signs are measured to help identify those infants at risk, or deteriorating, so that additional care can be provided. However, we would recommend that more extensive data be collected to refine the tool before deployment in the clinical environment.

## Conclusions

Vital signs are often excluded from analyses investigating risk factors associated with neonatal mortality. This study shows the importance of including these, along with any underlying conditions, as well as adding them to local guidelines concerning neonatal care. Low birthweight and temperature are an especially strongly associated with adverse outcomes, suggesting that greater use of incubators or kangaroo mother care could be useful [36]. We report that GLMs are superior to decision trees when assessing risk factors associated with mortality, and that ROC curves are useful tools for identifying cut-offs for clinical guidelines. This study shows how to combine multiple risk factors, including vital signs, from a GLM into a graphical tool that could be used in various low-resource settings to provide early warning of risk of mortality for infants in the first 28 days of life.

## Data Availability

The dataset analysed during the current study can be made available from the corresponding author to researchers on reasonable request.

## References

[1] Lissauer T, Fanaroff AA, Miall L, Fanaroff JM (2020). Neonatology at a glance.

[2] World Health Organisation (2019). Newborns: improving survival and well-being [internet]. WHO fact sheets.

[3] Afnan-Holmes H, Magoma M, John T, Levira F, Msemo G, Armstrong CE (2015). Tanzania’s countdown to 2015: an analysis of two decades of progress and gaps for reproductive, maternal, newborn, and child health, to inform priorities for post-2015. Lancet Glob Health.

[4] Mbawala G, Fredrick F, Kamugisha E, Konje E, Hokororo A (2014). Factors associated with mortality among premature babies admitted at Bugando medical Centre, Mwanza - Tanzania. East Afr J Public Health.

[5] Yamey G (2012). What are the barriers to scaling up health interventions in low and middle income countries? A qualitative study of academic leaders in implementation science. Glob Health.

[6] Lawn JE, Cousens S, Zupan J (2005). 4 million neonatal deaths: when? Where? Why?. Lancet.

[7] UNICEF. Every child alive: the urgent need to end newborn deaths. Geneva; 2018. Available from: https://data.unicef.org/resources/every-child-alive-urgent-need-end-newborn-deaths/

[8] UNICEF (2015). Maternal and newborn health disparities: Tanzania [internet].

[9] Kidanto HL, Massawe SN, Nystrom L, Lindmark G (2006). Analysis of perinatal mortality at a teaching hospital in Dar Es Salaam, Tanzania, 1999-2003. Afr J Reprod Health.

[10] Mmbaga BT, Lie RT, Olomi R, Mahande MJ, Kvale G, Daltveit AK (2012). Cause-specific neonatal mortality in a neonatal care unit in northern Tanzania: a registry based cohort study. BMC Pediatr.

[11] Duncan KD, McMullan C, Mills BM (2012). Early warning systems: the next level of rapid response. Nursing (Lond).

[12] Mitchell EJ, Qureshi ZP, Were F, Daniels J, Gwako G, Osoti A (2020). Feasibility of using an early warning score for preterm or low birthweight infants in a low-resource setting: results of a mixed-methods study at a national referral hospital in Kenya. BMJ Open.

[13] Mortensen N, Augustsson JH, Ulriksen J, Hinna UT, Schmölzer GM, Solevåg AL (2017). Early warning- and track and trigger systems for newborn infants: a review. J Child Health Care.

[14] Ministry of Health, Community Development, Gender, Elderly and Children (2019). National Guideline for Neonatal Care and Establishment of Neonatal Care Unit.

[15] World Health Organization, Department of Maternal Newborn Child and Adolescent Health (2013). WHO recommendations on postnatal care of the mother and newborn [internet].

[16] Jones RE, Lopez KH (2014). The neonate and the new parents. Human reproductive biology.

[17] Sutton RT, Pincock D, Baumgart DC, Sadowski DC, Fedorak RN, Kroeker KI (2020). An overview of clinical decision support systems: benefits, risks, and strategies for success. Npj Digit Med.

[18] Malhotra A, Rachet B, Bonaventure A, Pereira SP, Woods LM (2021). Can we screen for pancreatic cancer? Identifying a sub-population of patients at high risk of subsequent diagnosis using machine learning techniques applied to primary care data. Real FX, editor. PLoS One.

[19] Oonsivilai M, Mo Y, Luangasanatip N, Lubell Y, Miliya T, Tan P (2018). Using machine learning to guide targeted and locally-tailored empiric antibiotic prescribing in a children’s hospital in Cambodia. Wellcome Open Res.

[20] Peiffer-Smadja N, Rawson TM, Ahmad R, Buchard A, Georgiou P, Lescure F-X (2020). Machine learning for clinical decision support in infectious diseases: a narrative review of current applications. Clin Microbiol Infect.

[21] Roth JA, Battegay M, Juchler F, Vogt JE, Widmer AF (2018). Introduction to machine learning in digital healthcare epidemiology. Infect Control Hosp Epidemiol.

[22] The WHO young infants study group. Methodology for a multicenter study of serious infections in young infants in developing countries. Pediatr Infect Dis J. 1999;18(10) Available from: https://journals.lww.com/pidj/Fulltext/1999/10001/Methodology_for_a_multicenter_study_of_serious.3.aspx. 10.1097/00006454-199910001-00003 10530568

[23] World Health Organisation (2018). Preterm birth.

[24] Leduc D, Woods S (2000). Community Paediatrics committee. Temperature measurement in paediatrics. Paediatr Child Health.

[25] Therneau T, Atkinson B (2019). Recursive partitioning and regression tree [internet].

[26] Sing T (2020). Package ‘ROCR’.

[27] World Health Organization. Kangaroo mother care: a practical guide. Geneva; 2003. p. 48. Available from: https://www.who.int/publications/i/item/9241590351

[28] Arunda MO, Agardh A, Asamoah BO (2018). Survival of low birthweight neonates in Uganda: analysis of progress between 1995 and 2011. BMC Pregnancy Childbirth.

[29] Kumar V, Shearer JC, Kumar A, Darmstadt GL (2009). Neonatal hypothermia in low resource settings: a review. J Perinatol.

[30] Welaga P, Moyer CA, Aborigo R, Adongo P, Williams J, Hodgson A (2013). Why are babies dying in the first month after birth? A 7-year study of neonatal mortality in northern Ghana. Moormann AM, editor. PLoS ONE.

[31] Davidson R, Brent A, Seale A (2014). Oxford handbook of tropical medicine.

[32] Cavallin F, Menga A, Brasili L, Maziku D, Azzimonti G, Putoto G (2020). Factors associated with mortality among asphyxiated newborns in a low-resource setting. J Matern-Fetal Neonatal Med Off J Eur Assoc Perinat Med Fed Asia Ocean Perinat Soc Int Soc Perinat Obstet.

[33] Floares A, Birlutiu A (2012). Decision tree models for developing molecular classifiers for cancer diagnosis. The 2012 international joint conference on neural networks (IJCNN).

[34] Surucu M, Shah KK, Mescioglu I, Roeske JC, Small W, Choi M (2016). Decision trees predicting tumor shrinkage for head and neck cancer: implications for adaptive radiotherapy. Technol Cancer Res Treat.

[35] Ghiasi MM, Zendehboudi S (2021). Application of decision tree-based ensemble learning in the classification of breast cancer. Comput Biol Med.

[36] Winkler LA, Noon S, Babwanga T (2018). A multi-year project implementing kangaroo mother care in rural Tanzania. Am J Trop Med Hyg.

